# Heavy Metal Contamination of Foods by Refuse Dump Sites in Awka, Southeastern Nigeria

**DOI:** 10.1100/tsw.2008.129

**Published:** 2008-10-01

**Authors:** J. K. C. Nduka, O. E. Orisakwe, L. O. Ezenweke, M. N. Chendo, T. E. Ezenwa

**Affiliations:** ^1^ Pure and Industrial Chemistry Department, Nnamdi Azikiwe University, Awka, Anambra State, Nigeria; ^2^ Toxicology Unit, Pharmacology Department College of Health Sciences, Nnamdi Azikiwe University, Nnewi Campus, Nnewi, Anambra State, Nigeria; ^3^ Chemistry Department, Anambra State University, Uli, Nigeria

**Keywords:** heavy metals, refuse dumps, farm produce, environmental pollution, Nigeria

## Abstract

The impact of heavy metals from refuse dumps on soil, food, and water qualities in Awka, Nigeria was studied. Soil samples (top and 1.35 m deep) were collected from five refuse dumps digested with conc. HNO_3_ and HClO_4_. The heavy metals (lead, manganese, arsenic, chromium, cadmium, and nickel) in vegetables (spinach, fluted pumpkin), root crop (cocoyam), and surface and ground water were determined using an atomic absorption spectrophotometer (AAS). Chemical properties of the soil and bacteria were determined. Heavy metals were found to be more concentrated at a depth of 1.35 m. Manganese was high in shallow wells and borehole water samples with the highest levels as 0.538 and 0.325 mg/l, respectively. Nickel levels in the borehole sample ranged from 0.001 to 0.227 mg/l, whereas the highest level of lead was 0.01 mg/l. The Obibia stream had the highest levels of manganese and lead. Linear regression analyses showed that the relationship between soil heavy metals and farm produce heavy metals was strong. Taken together, we may conclude that the consumption of leafy vegetables and crops produced on contaminated soils may pose a health risk to those that reside around the refuse dumps.

## INTRODUCTION

Plant uptake is one of the major pathways by which heavy metals in soils enter the human food chain[1]. Food crops such as cassava, cocoyam, and other tuber crops grown in mining communities take up toxic or hazardous chemicals, such as arsenic and cadmium, from the soil. This study evaluated the human health risk from eating cassava grown in some mining communities in Ghana[2]. Also, concerns over the possible increase in phytoavailability of biosolids-applied trace metals to plants have been raised based on the assumption that decomposition of applied organic matter would increase phytoavailability[3]. It has been found that uptake of gaseous mercury was the predominant pathway by which the mercury accumulated in the foliage, and mercury in root tissue is mainly derived from soil[4]. Cadmium pollution of soil affects the soil-plant pathway[1]. Shute and Macfie's work on the cadmium and zinc accumulation in soybeans reinforces the need to monitor concentrations of toxic metals in food crops[5]. The uptake of heavy metals by lettuce and mustard rape in sewage sludge and effluent-exposed soil suggests significant uptake of the heavy metals from the soil[6].

Our previous study[7] suggested that refuse dumps can substantially increase the environmental burden of heavy metals in Awka municipality, Nigeria. Soil contamination may adversely impact human health when agricultural produce grown in and around dump sites are contaminated, or when infiltration and surface runoff contribute to ground and surface water contamination. Because organic and inorganic compounds that contain heavy metals are common at refuse dump sites in Awka, they degrade and decompose following favorable weather conditions, thereby releasing various pollutants into the soil and ground water.

In this follow-up study, we investigated if heavy metal levels of farm produce harvested from refuse dump sites in Awka positively correlated with soil samples in the same locations. Heavy metal levels of ground and surface water within the study areas, changes in soil properties, and bacterial isolates are also presented.

## METHODS

We sampled the five biggest refuse dump sites in Awka (Fig. 1, labeled sites A–E). These sites are mainly used for the dumping of domestic and sundry refuse. All the soil samples are fine to medium sand in texture. For consistency across sites, two soil samples were collected in ring form at the surface and at a depth of 1.35 m, and stored in black polyethylene bags before digestion and analysis. The temperature of the laboratory was 29.5°C +/- (ambient temperature). Samples were ground, mixed, and divided into fine particles that could pass through a 0.5-mm sieve. Soil samples were digested by adding 2 g of soil to 15 ml of concentrated nitric acid and perchloric acid at a ratio 1:1, and allowed to stand for 135 min until the mixture became colorless. The samples were filtered and washed with 15 ml of deionized water, and made up the filtrate to 100 ml in a standard flask. Six heavy metals (lead, manganese, arsenic, chromium, cadmium, and nickel) were determined in the filtrate at their respective wavelengths using an atomic absorption spectrophotometer (AAS) 200A[7]. Heavy metals of surface and ground water were also determined using clean filtrates of the samples. The heavy metal levels of food samples, such as vegetables (spinach [*Amanthus hydbridus*], fluted pumpkin [*Telfairia occidentals*]) and root crop (cocoyam [*Xanthosoma sagittifolium*]), were determined after ashing and digestion. Linear regression and correlation coefficient between soil heavy metals and farm produce heavy metals were calculated.

Percentage organic matter (carbon) of surface soil at refuse dumps was determined according to Emedo et al.[8]. Exchangeable cations, such as potassium, sodium, and calcium, were determined on a flame photometer, while magnesium was determined with AAS. Bulk density, particle density, percentage porosity, and cation exchange capacity were determined[9]. Soil pH was determined by weighing 20 g of air-dried soil (passing through a 2-mm sieve) into a 50-ml beaker, then adding 20 ml of distilled water, allowing to stand for 30 min, while stirring occasionally with a glass rod. An electrode of the pH meter was inserted into the partly settled suspension and pH measured; the result was reported as "soil pH in water". For bacterial isolates, soil samples were collected in sterile specimen bottles from a depth of 0.05 m after scrapping the surface debris, and treated within 90 min. The samples were air dried and sieved using a wire mesh. About 10 g of the sieve samples was mixed in 10 ml of normal saline and serially diluted up to $10^5$, then 0.1 m of the sample was aseptically spread on freshly prepared nutrient agar plates, and incubated at 37°C for 24 h. The plates were inverted, the colonies were subcultured onto freshly prepared nutrient agar plates, and incubated at 37°C for 24 h. From the pure cultures, standard characterization tests, including gram stain, motility, and fermentation were carried out. The identification of the isolates was based on the method of Cruickshank et al.[10] and Holt[11]. Control samples were collected 100 m from the dump sites. In all, the triplicate samples agreed very well. The result given is the mean of three estimations and was analyzed statistically using the Student t-test.

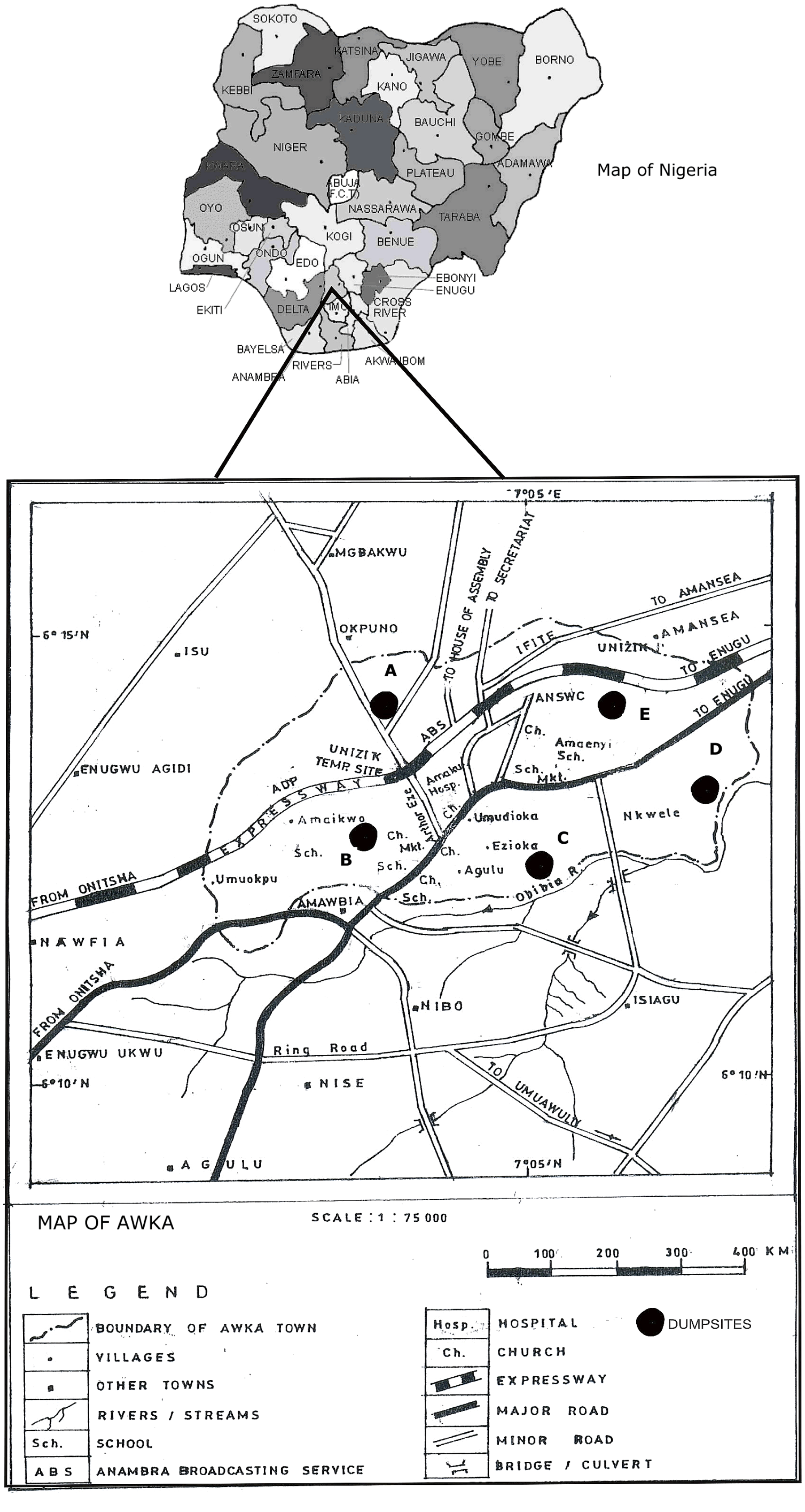

**FIGURE 1.** Map of Awka.

## RESULTS

Results indicate the presence of all the metals assessed. There were high levels of chromium, manganese, and nickel at the surface of site A. Lead and cadmium were more at the surface of site B; manganese and cadmium were found to be more at the surface of site C; lead, arsenic, chromium, and cadmium at the surface of site D. Whereas manganese, arsenic, chromium, and nickel were found to be more at the surface of site E, most of these heavy metals were found to be more at a depth of 1.35 m (see Table 1). In all, only chromium showed a significant difference ($p < 0.05$) at the 1.35-m depth.

**TABLE 1**
**Metal Levels in mg/kg of Soil at Different Dump Sites**

| | Depth (m) | Pb | Mn | As | Cr | Cd | Ni |
|---|---|---|---|---|---|---|---|
| Site A | 0.00 | 350 (83.8) | 290 | 620 (ND) | 4.75 | 2.00 | 27.00 (ND) |
| | 1.35 | 510 | 280.5 | 350 | 4.00 | 3.00 | 21.00 |
| Site B | 0.00 | 500 (ND) | 135 | 30 (ND) | 2.17 | 2.85 | 18.95 (ND) |
| | 1.35 | 350 | 160 | 65 | 2.50 | 2.38 | 31.50 |
| Site C | 0.00 | 620 (ND) | 120 | 102 (ND) | 2.35 | 2.66 | 15.00 (ND) |
| | 1.35 | 613.50 | 105 | ND | 2.43 | 2.57 | 18.00 |
| Site D | 0.00 | 401 (3.80) | 55.70 | 31.85 (ND) | 2.63 | 2.80 | 64.50 (ND) |
| | 1.35 | 370 | 60.55 | ND | 2.62 | 1.79 | 68.35 |
| Site E | 0.00 | 145.30 (ND) | 205 | 160 (ND) | 3.09 | 2.14 | 61.75 (ND) |
| | 1.35 | 160.30 | 136.85 | 65.90 | 2.43 | 3.15 | 46.50 |

ND = Not detected, control values are in parentheses.

Table 2 shows the concentration of heavy metals (mg/l) of shallow/hand dug (S) and borehole (B) well water. Manganese was found to be high in both shallow/hand dug and borehole water samples with highest levels as 0.538 and 0.325 mg/l, respectively. Nickel levels in the borehole sample ranged from 0.001 to 0.227 mg/l, whereas the highest level of lead was 0.01 mg/l. The concentrations of metals in nearby streams, namely Okpuno, Ezu, and Obibia, are shown on Table 3. Obibia had the highest levels of heavy metals, 1.91 and 0.01 mg/l of manganese and lead, respectively.

Table 4 shows the heavy metal levels in food samples, such as vegetables (spinach [*Amanthus hydbridus*], fluted pumpkin [*Telfairia occidentals*]) and root crop (cocoyam [*Xanthosoma sagittifolium*]). With the exception of spinach, all the food samples contained measurable levels of iron, lead, manganese, arsenic, chromium, cadmium, and nickel; fluted pumpkin contained the highest levels of these heavy metals.

Linear regression analyses showed that the relationship between soil heavy metals and farm produce heavy metals was strong.

Chemical tests show the presence of exchangeable cation and exchange capacity in an amount that will make nutrients available to plants (Table 5). Bacteria isolates from the dump sites are shown on Table 6. *Staphylococcus aureus, Pseudomonas* sp., *Streptococcus* sp; *Escherichia coli,* and *Proteus* sp. were seen at all the dump sites. *Bacillus* sp. and *Klebsiella* sp. were not detected in dump sites B and C.

## DISCUSSION

Accumulation of heavy metals by crop plants from contaminated soils may pose health risks[12]. Plant shoots have been found to be contaminated by heavy metals, particularly cadmium, chromium, nickel, and

**TABLE 2**
**Concentration of Heavy Metals (mg/l) of Shallow/Hand Dug (S) and Borehole (B) Well Water**

| Pb (S) | Pb (B) | Mn (S) | Mn (B) | As (S) | As (B) | Cr (S) | Cr (B) | Cd (S) | Cd (B) | Ni (S) | Ni (B) |
|---|---|---|---|---|---|---|---|---|---|---|---|
| ND | 0.008 | 0.250 | 0.271 | ND | ND | ND | ND | ND | 0.005 | 0.002 | 0.227 |
| ND | ND | 0.101 | 0.325 | — | — | — | — | ND | 0.001 | ND | 0.008 |
| ND | ND | ND | 0.121 | — | — | — | — | ND | ND | ND | ND |
| ND | 0.008 | 0.385 | 0.072 | — | — | — | — | ND | ND | 0.005 | 0.005 |
| 0.008 | ND | 0.173 | 0.037 | — | — | — | — | 0.002 | 0.001 | ND | 0.008 |
| 0.008 | ND | 0.084 | 0.167 | — | — | — | — | 0.003 | 0.005 | ND | ND |
| ND | ND | 0.036 | 0.065 | — | — | — | — | ND | 0.001 | 0.008 | ND |
| ND | 0.01 | 0.538 | 0.03 | — | 0.01 | — | 0.01 | 0.001 | 0.001 | 0.016 | 0.001 |
| 0.008 | 0.01 | 0.005 | 0.03 | — | 0.01 | — | 0.001 | 0.005 | 0.001 | ND | 0.001 |
| — | 0.01 | — | 0.07 | — | 0.01 | — | 0.001 | — | 0.001 | — | 0.001 |
| — | 0.01 | — | 0.05 | — | 0.01 | — | 0.001 | — | 0.001 | — | 0.001 |

ND = Not detected.

**TABLE 3**
**Concentration of Metals (mg/l) in Nearby Streams**

| Name of Stream | Pb | Mn | As | Cr | Cd | Ni |
|---|---|---|---|---|---|---|
| Okpuno | ND | 0.015 | ND | ND | 0.003 | ND |
| Ezu | 0.001 | 0.02 | 0.001 | 0.001 | 0.001 | 0.001 |
| Obibia | 0.01 | 1.91 | 0.001 | 0.01 | 0.01 | 0.03 |

ND = Not detected.

**TABLE 4**
**Concentration of Metals in mg/kg of Food Harvested from Dump Sites**

| Food Type | Pb | Mn | As | Cr | Cd | Ni |
|---|---|---|---|---|---|---|
| Spinach (*Amanthus hydbridus*) | 1.30 | 9.67 | ND | 3.88 | 0.67 | 1.06 |
| Fluted pumpkin (*Telfairia occidentals*) | 1.74 | 20.57 | 0.10 | 0.10 | 1.55 | 1.65 |
| Cocoyam (*Xanthosoma sagittifolium*) | 0.10 | 0.10 | 0.10 | 0.01 | 0.05 | 0.77 |

ND = Not detected.

lead, in amounts that exceed the guidance limits set by State Environmental Protection Protection (SEPA), China and the World Health Organization[12]. The present study investigated the environmental impact of the refuse dump sites with a capture of the heavy metal levels in potable water and food samples — like spinach (*Amanthus hydbridus*), fluted pumpkin (*Telfairia occidentals*), and cocoyam (*Xanthosoma sagittifolium*), which form the staple food of the local population.

**TABLE 5**
**Chemical Properties of Surface Soil at the Dump Sites**

| Surface Soil Sample of Different Sites | Parameters* | | | | | |
|---|---|---|---|---|---|---|
| | Cation Exchange Capacity ($cmol_c$/kg) | Particle Density ($mg/m^3$) | Bulk Density ($mg/m^3$) | pH | % Pore Space | % Organic Matter |
| Site A | 7.66 (3.32) | 2.55 (2.65) | 1.78 (1.73) | 6.61 (6.22) | 30.20 (34.72) | 2.55 (0.00) |
| Site B | 6.16 (4.04) | 2.50 (2.70) | 1.88 (1.83) | 6.62 (4.45) | 24.80 (32.22) | 1.72 (0.79) |
| Site C | 5.92 (3.16) | 2.60 (2.75) | 1.83 (1.73) | 6.69 (5.26) | 29.62 (37.09) | 1.45 (0.00) |
| Site D | 6.56 (4.24) | 2.80 (2.55) | 1.83 (1.78) | 6.68 (6.38) | 34.64 (30.20) | 2.10 (0.86) |
| Site E | 7.48 (3.04) | 2.75 (2.65) | 1.68 (1.73) | 6.60 (6.65) | 38.90 (34.72) | 3.07 (0.00) |

* Control values in parentheses.

**TABLE 6**
**Bacteria Isolates from the Dump Sites**

| Bacteria | Site A | Site B | Site C | Site D | Site E |
|---|---|---|---|---|---|
| *Staphylococcus aureus* | Present | Present | Present | Present | Present |
| *Pseudomonas* sp. | Present | Present | Present | Present | Present |
| *Streptococcus* sp. | Present | Present | Present | Present | Present |
| *Escherichia coli* | Present | Present | Present | Present | Present |
| *Bacillus* sp. | Present | Absent | Absent | Present | Present |
| *Klebsiella* sp. | Present | Absent | Absent | Present | Present |
| *Proteus* sp. | Present | Present | Present | Present | Present |

Heavy metal levels as in our previous study[7] were found to be higher at the 1.35-m depth. Soil porosity, a shallow water table, and high annual rainfall facilitate rapid leaching and infiltration into the underground water. In part, this may explain the high level of some of these metals at the 1.35-m depth. The high content of these metals is less surprising, as some are associated with organic compounds that are common at the dump sites. Manganese was found to be high in both shallow/hand dug and borehole well water samples, with the highest levels as 0.538 and 0.325 mg/l, respectively. Nickel levels in the borehole sample ranged from 0.001 to 0.227 mg/l, whereas the highest level of lead was 0.01 mg/l. The Obibia stream had the highest levels of heavy metals, 1.91 and 0.01 mg/l of manganese and lead, respectively. The levels of these heavy metals were below the U.S. Environmental Protection Agency's maximum contaminant level (USEPA MCL; MCL for lead is 0.015 mg/l, that of cadmium is 0.005 mg/l).

With the exception of spinach, all the food samples contained measurable levels of lead, manganese, arsenic, chromium, cadmium, and nickel. Fluted pumpkin contained the highest levels of these heavy metals. The entry of these heavy metals into the food chain has important public health implications. In 2004, Orisakwe and coworkers[13] reported high levels of lead in aerial fruits near an industrial area in Nigeria.

Chemical tests revealed the presence of exchangeable cations and exchange capacity in an amount that will make nutrients available to plants. Cation exchange capacity (the sum total of the exchangeable cations that a soil can adsorb) is a function of a variety of soils and soil materials; humus soil (with high organic matter) has high cation exchange capacity (CEC). This is evident in our results and is also supported by literature[9]. A combination of biological processes (decomposition and accumulation), carbon (iv) oxide ($CO_2$), methane ($CH_4$), and nitrous oxide ($N_2O$) are generated. The cation exchange of most soils increases with pH. At very low pH values, the CEC is also generally low; as the pH is raised (alkalinity), CECs increase. To obtain a measure of this maximum retentive capacity, the CEC is commonly determined at pH 7 or above. By cation exchange, nutrient cations are forced into soil solutions where they are available for root hairs. The acidity of the top soil at dump sites and that of the control does not vary much because of high exchangeable cations, such as sodium, magnesium, calcium, and potassium, previously reported from our laboratory[7], which are not highly leachable (when leached, they are replaced by hydrogen ion) because most components of the studied dump sites have been found to contain high levels of these metals[14].

In their studies that evaluated cancer and noncancer health effects from eating cassava grown in the mining areas of Ghana in accordance with the USEPA Risk Assessment guidelines, Obiri et al.[2] found cancer health risk values above the acceptable cancer risk range. Tandi et al.[6] concluded that long-term addition of sewage sludge/effluent to farm soils increased the concentration of zinc and copper in top soil to levels that pose environmental concern. Our study is in agreement with the work of Khan and coworkers[12] that there may be potential health risks associated with cultivation and consumption of crops and vegetables grown on contaminated soils. The soil uptake of the heavy metals may probably be related to the differences in organic matter of the soils.

Heavy metals in food samples were positively correlated with soil levels indicating soil contamination. Although the present study implicates soil contamination, we also suggest further studies to investigate diffuse pollution by aerial deposition due to the presence of smog in the city. Taken together and in comparison with other studies, we may conclude that the consumption of leafy vegetables and root crops produced on contaminated soils poses a health risk to poor communities that reside around the refuse dump sites.

*Staphylococcus aureus, Pseudomonas* sp., *Streptococcus* sp., *Escherichia coli, Bacillus* sp., *Klebsiella* sp., and *Proteus* sp. were isolated from all the dump sites, except sites B and C where *Bacillus* and *Klebsiella* were not found. These bacteria have been variously reported in dump site studies. *E. coli* and *Streptococcus* sp. have been reported by Ekundayo[15], while *Bacillus* sp., *E. coli, Pseudomonas* sp., and *Klebsiella* sp. have been reported by Cook et al.[16]. *Pseudomonas* sp. has been widely reported to be associated with waste[17]. All the bacteria isolated have been reported as potential pathogens by Cook et al.[16].

Since the dumps are within residential areas, and there are no landfills in Awka and in most Nigerian cities, domestic animals and nocturnal insects can serve as vectors of these pathogens. Whereas the refuse dump sites may not be very important in the overall heavy metal pollution of the potable water supply, it is evidenced in the present study that refuse dump sites impact positively on the heavy metal contamination of food samples like vegetables, such as spinach (*Amanthus hydbridus*) and fluted pumpkin (*Telfairia occidentals*), and root crops, such as cocoyam (*Xanthosoma sagittifolium*), in Awka, Nigeria. This entry and eventual contamination of the food chain has caused a serious public health concern.
